# NeoPAIR-T: Functional Mapping of Neoantigen–TCR Pairs Using a CRISPR-Engineered Jurkat Reporter System

**DOI:** 10.3390/cells14221789

**Published:** 2025-11-14

**Authors:** Koji Nagaoka, Yukari Kobayashi, Kazuhiro Kakimi

**Affiliations:** Department of Immunology, Kindai University Faculty of Medicine, Sakai 590-0197, Osaka, Japan; knagaoka@med.kindai.ac.jp (K.N.); yukkoba@med.kindai.ac.jp (Y.K.)

**Keywords:** neoantigen, T-cell receptor, functional screening, CRISPR/Cas9, personalized immunotherapy, single-cell analysis, tandem minigene

## Abstract

**Highlights:**

**What are the main findings?**

- A CRISPR/Cas9-engineered Jurkat reporter system (NeoPAIR-T) was developed for multiplexed, functional identification of authentic neoantigen–TCR pairs.
- The NeoPAIR-T platform integrates transcriptome-guided TCR selection with tandem minigene-based antigen presentation, enabling parallel screening and precise mapping of tumor-reactive TCRs.

**What are the implications of the main findings?**

- NeoPAIR-T bridges the gap between computational prediction and functional validation, providing a workflow designed for scalability in neoantigen–TCR discovery.
- This platform offers a practical foundation for accelerating the development of personalized cancer immunotherapies such as neoantigen vaccines and TCR–T cell therapy.

**Abstract:**

Targeting mutation-derived neoantigens is a promising strategy for personalized immunotherapies. However, identifying true neoantigens and cognate T cell receptors (TCRs) remains challenging because computational prediction of neoantigen peptides is uncertain and most tumor-infiltrating lymphocytes are bystanders rather than tumor-reactive, necessitating functional validation. Here, we developed NeoPAIR-T (Neoantigen–TCR Pairing Assay using reporter T cells), a functional assay based on co-culture of TCR–T reporter cells and autologous antigen-presenting cells (APCs) to screen neoantigen–TCR pairs. Reporter T cells are Jurkat-derived cells engineered to express a luciferase/eGFP dual reporter, providing quantitative readouts of TCR activation, while APCs are immortalized autologous cells transfected with tandem minigenes (TMGs) encoding predicted neoantigens, bypassing peptide synthesis. NeoPAIR-T also includes TCRα-knockout with targeted knock-in of candidate TCRs at the TCRβ locus to prevent mispairing and enables parallel testing of multiple reporter T cell clones co-cultured with the same APCs for efficient identification of functional pairs. Using lung cancer samples, whole-exome and RNA sequencing predicted 63 candidate peptides assembled into three TMGs. Single-cell RNA/TCR sequencing identified eight TCR clonotypes, introduced into reporter T cells and tested in parallel. Co-culture with TMG-expressing APCs revealed two functional neoantigen–TCR pairs validated by peptide assays (EC_50_: 10^−9.2^–10^−6.7^ M). Collectively, NeoPAIR-T streamlines neoantigen–TCR identification for vaccine and TCR-T applications.

## 1. Introduction

Neoantigens, which arise from tumor-specific somatic mutations and are absent in normal tissues, have emerged as ideal targets for personalized cancer immunotherapy [1]. Because they are recognized exclusively as “non-self” antigens, neoantigens can elicit highly tumor-specific T-cell responses without inducing autoimmunity. Accordingly, neoantigen-based vaccines and T cell receptor-engineered T cell (TCR-T) therapies have shown encouraging results in early clinical trials [2,3,4,5,6,7]. These strategies provide a promising avenue for the development of safe and effective cancer immunotherapies tailored to individual patients. Indeed, the clinical success of these approaches, particularly in combination with immune checkpoint inhibitors, underscores that expanding the tumor-specific T cells represents a key strategy to synergize with and augment the clinical benefit of existing immunotherapies [8].

However, the realization of neoantigen-targeted immunotherapies still faces major challenges in both antigen and TCR discovery. On the antigen discovery side, while in silico prediction algorithms have improved, they still generate a substantial number of false-positive candidates that fail to elicit T-cell activation [9,10]. On the TCR discovery side, the challenge is that the tumor is infiltrated by a vast population of bystander T cells, among which the rare, truly specific TCRs must be identified [11]. Thus, identifying which neoantigens are genuinely recognized by T cells in vivo remains elusive. To overcome this limitation, functional assays capable of simultaneously identifying both neoantigens and their cognate T cells (or their TCRs) are required.

Recent advances in single-cell sequencing have made it possible to determine the functional state of each T cell—such as activation, exhaustion, or differentiation—based on its transcriptomic profile, thereby enabling the prediction of neoantigen-specific T cells through their characteristic functional phenotypes [12,13,14,15]. Consequently, researchers are now confronted with the formidable task of validating which of the many transcriptome-prioritized TCRs can truly recognize a genuine neoantigen within an equally extensive and error-prone pool of in silico predictions [16,17].

In current practice, identification of bona fide neoantigens still relies on labor-intensive experimental validation. For each patient, somatic mutation lists and HLA typing data are used to predict potential MHC-binding peptides, which are then chemically synthesized. The synthesized peptides are pulsed onto antigen-presenting cells (APCs) expressing the patient’s HLA molecules and co-cultured with patient-derived peripheral blood mononuclear cells (PBMCs) or tumor-infiltrating lymphocytes (TILs) to assess antigen-specific T-cell responses. However, this validation process remains technically challenging and low throughput due to several factors: (1) the need to synthesize and test a large number of candidate peptides, which is both laborious and costly; (2) the requirement for HLA-matched APCs; (3) the limited availability of PBMCs and TILs from clinical specimens; and (4) potential biases introduced during in vitro expansion of TILs, which may not accurately reflect their ex vivo reactivity. These limitations hinder the efficient translation of neoantigen discoveries into clinical applications.

To overcome these obstacles, we developed NeoPAIR-T (Neoantigen–TCR Pairing Assay using reporter T cells), a patient-specific, multiplexed system for the parallel identification of true neoantigens and their cognate TCRs. NeoPAIR-T uniquely integrates four key features that enable multiplexed and quantitative functional screening: (i) a luciferase/eGFP dual-reporter for sensitive, quantitative assessment of TCR activation; (ii) a TCRα-knockout combined with targeted knock-in of candidate TCRαβ into the endogenous TCRβ CDR3 region, preventing TCR mispairing and ensuring physiological expression; (iii) Epstein–Barr virus (EBV)-immortalized autologous B-cell APCs combined with tandem minigene (TMG) libraries, which offer a practical and widely used platform for presenting large numbers of candidate neoantigens in vitro, while still differing in aspects of antigen processing and HLA presentation compared with autologous tumor cells; and (iv) parallelized testing of multiple TCR–antigen pairs. This optimized NeoPAIR-T system enables efficient, multiplexed functional validation of predicted neoantigens and their specific TCRs in a physiologically relevant context.

## 2. Materials and Methods

### 2.1. Cell Lines, Culture Conditions, and Reagents

Jurkat E6-1 cells (TIB-152, ATCC, Manassas, VA, USA) and all derivative lines were maintained in RPMI-1640 medium (FujiFilm Wako Pure Chemical Corporation, Osaka, Japan) supplemented with 10% fetal bovine serum (FBS, Sigma-Aldrich, St. Louis, MO, USA) and 1% penicillin-streptomycin (Nacalai Tesque, Kyoto, Japan) under a humidified atmosphere of 5% CO_2_ at 37 °C. 293FT cells (Thermo Fisher Scientific, Waltham, MA, USA) were maintained in DMEM (high glucose, FujiFilm Wako Pure Chemical Corporation) supplemented with 10% FBS and 1% penicillin-streptomycin. Epstein–Barr virus-transformed lymphoblastoid cell lines (EBV-LCLs) were established by infecting PBMCs with the B95-8 supernatant and maintained in RPMI-1640 medium supplemented with 20% FBS and 1% penicillin-streptomycin. Jurkat E6-1 and EBV-LCL tested negative for mycoplasma by a PCR-based assay. Experiments were performed using early-passage cells (≤10 passages after thawing). A comprehensive list of reagents used in this study is provided in Table S1.

### 2.2. Preparation of Guide RNA (gRNA) and Ribonucleoprotein (RNP) Complexes

The target sites for all CRISPR RNAs (crRNAs) are depicted in Figure S1A and the crRNA sequences are listed in Table S2 [18]. Alt-R crRNAs and Alt-R tracrRNA were purchased from Integrated DNA Technologies (IDT, Coralville, IA, USA) and reconstituted in Nuclease-Free Duplex Buffer (IDT) to a final concentration of 100 µM. To form the gRNA, equimolar amounts of crRNA and trans-activating crRNA (tracrRNA) were combined, heated to 95 °C for 5 min and then annealed by incubating at room temperature for 10 min. For RNP assembly, 3 μL (150 pmol) of the prepared gRNA was gently mixed with approximately 2 μL (60 pmol) of TrueCut Cas9 Protein v2 (Thermo Fisher Scientific) and incubated at room temperature for 10 min to allow for complex formation.

### 2.3. Lentivirus Production

A DNA construct encoding human CD8α and CD8β, linked by a T2A self-cleaving peptide sequence (human CD8α-T2A-CD8β), was synthesized by IDT. This synthesized gene was subsequently cloned into the pLenti-III-EF1alpha lentiviral vector (Figure S1B, Applied Biological Materials Inc., Richmond, BC, Canada).

For lentivirus production, 293FT cells were co-transfected with the pLenti-III-EF1alpha-human CD8α-T2A-CD8β plasmid and the ViraPower Lentiviral Packaging Mix (Thermo Fisher Scientific) using Lipofectamine 2000 (Thermo Fisher Scientific) according to the manufacturer’s protocol. The virus-containing supernatant was harvested at 48 h post-transfection. The supernatant was centrifuged to remove cell debris and then concentrated using the Peg-It Lentivirus Concentration Reagent (System Biosciences, LLC, Palo Alto, CA, USA) according to the manufacturer’s protocol. The resulting viral pellet was resuspended in RPMI-1640 medium at 1/10th of the original volume, aliquoted, and stored at −80 °C until use.

### 2.4. Establishment of the Reporter T Cell Line

The parental Jurkat E6-1 cells were characterized by flow cytometry as CD3^+^, CD4^+^, CD8^−^, TCRαβ^+^, and FAS^+^. A series of genetic modifications were performed as follows to establish the reporter T cell line.

First, the CD4 and FAS genes were sequentially disrupted (Figure S1). To knock out each gene, 4 × 10^5^ cells were washed with DPBS and resuspended in 20 µL of SE Cell Line Nucleofector Solution (Lonza, Basel, Switzerland). A preassembled RNP complex (5 µL) and 0.8 µL of Alt-R Cas9 Electroporation Enhancer (IDT) were added to the cell suspension. Electroporation was performed using program CL-120 on the 4D-Nucleofector System (Lonza). Immediately after electroporation, the cells were transferred to prewarmed culture medium. The CD4 gene was knocked out first, and the CD4-negative population was isolated by cell sorting on an SH800S cell sorter (Sony Corporation, Tokyo, Japan). Subsequently, the FAS gene was knocked out in these cells using the same procedure, followed by cell sorting to isolate the CD4^−^FAS^−^ population.

Next, the CD4^−^FAS^−^ cells were transduced with the human CD8-expressing lentivirus in the presence of 6 µg/mL polybrene. Transduced cells were selected with 1 µg/mL puromycin (Thermo Fisher Scientific), and the CD8-positive population was subsequently sorted.

CD8-expressing cells were transduced with lentiCas9-Blast lentivirus (Figure S1B, Addgene #52962-LV, Addgene, Watertown, MA, USA) [19]. To knock out the T cell receptor alpha constant (TRAC) gene, these Cas9-expressing cells were electroporated (program CL-120) with a gRNA targeting TRAC (Table S2), without the addition of exogenous Cas9 protein. CD3-negative cells were then enriched by cell sorting.

Finally, these cells were transduced with a lentiviral NFAT-Luciferase-eGFP reporter construct (Figure S1B, BPS Bioscience, San Diego, CA, USA) at MOI of 20 and selected with 2 mg/mL G418 (FujiFilm Wako Pure Chemical Corporation). To enrich functionally responsive cells, the reporter T cells were stimulated overnight with 2 ng/mL PMA and 100 nM ionomycin (Sigma-Aldrich), followed by sorting for the eGFP-positive population.

We did not perform quantitative per locus indel-efficiency measurements or experimental off-target profiling. A formal stability study across passages was not conducted.

### 2.5. Patient and Clinical Samples

A patient with non-small cell lung carcinoma was included in this study after written informed consent had been obtained. Tumor tissue and peripheral blood samples were obtained from the patient. The study was conducted with the approval of the Institutional Review Boards of the Faculty of Medicine and Graduate School of Medicine of The University of Tokyo (G3545) and Kindai University Faculty of Medicine (R05-124).

### 2.6. Single-Cell RNA/TCR-Sequencing (RNA/TCR-Seq)

Fresh tumor tissue was obtained from one patient with lung squamous cell carcinoma (LK117) at the time of surgical resection. Tumor tissue was dissociated into a single-cell suspension using the Tumor Dissociation Kit, human, in combination with gentleMACS Dissociators (Miltenyi Biotec, Bergisch Gladbach, Germany) according to the manufacturer’s protocol. After staining the cells with FITC-anti-human CD8, Pacific Blue-anti-human CD4 antibodies (BioLegend, San Diego, CA, USA) and propidium iodide (Sigma-Aldrich), live (propidium iodide^−^) CD4^−^CD8^+^ T cells were then enriched by a SH800S cell sorter (Figure S2, Sony Corporation). Single-cell RNA-sequencing (RNA-Seq) combined with TCR repertoire analysis was performed using the Chromium Next GEM Single Cell 5′ v2 Reagent Kit (10x Genomics, Pleasanton, CA, USA) and Chromium Single Cell Human TCR Amplification Kit (10x Genomics) according to the manufacturer’s instructions. A total of 10,000 target cells were loaded for library construction. Libraries for both gene expression and paired TCRα/β (V(D)J) sequences were generated and pooled at a 9:1 ratio, followed by sequencing on an Illumina NovaSeq 6000 platform (Illumina, San Diego, CA, USA) to achieve a depth of approximately 50,000 read pairs per cell. Raw sequencing data were processed using Cell Ranger (10x Genomics, version 7.0.0) according to the manufacturer’s instructions. Reads were aligned to the GRCh38 human reference genome using the Cell Ranger pipeline, and gene expression matrices were generated after filtering and unique molecular identifier (UMI) counting. TCR V(D)J sequences were simultaneously assembled using the Cell Ranger V(D)J pipeline, yielding paired α and β chain clonotypes.

Single-cell RNA-Seq data were analyzed using Seurat (version 5.3.0) in R. Low-quality cells were removed by filtering out those with fewer than 200 or more than 5000 detected genes, or with mitochondrial transcript content exceeding 10%. The data were then log-normalized and scaled. V(D)J sequencing data were integrated using scRepertoire (version 1.12.0) [20], and only cells with both productive TCRα and TCRβ chains were retained for downstream analysis. The top 40 most frequent TCR clonotypes are listed in Table S4. CXCL13 expression scores were calculated from normalized and scaled expression data, and neoTCR8 signature scores were computed by single-sample gene set enrichment analysis (ssGSEA, GSVA package version 1.50.5) using normalized expression values based on the previously defined gene set reported by Lowery et al. [13].

### 2.7. Generation of Homology-Directed Repair (HDR) Templates

Eight candidate TCR sequences were obtained from the Loupe V(D)J Browser (version 5.0.1, 10x Genomics) and listed in Table S5. DNA cassettes containing TCRα-T2A-TCRβ sequences flanked by approximately 900 bp homology arms were synthesized by IDT. For multiplex screening, a fluorescent barcode–coding one of four fluorescent proteins (mCherry, mTagBFP2, Katushka, or moxCerulean3 [21,22])–together with P2A was inserted upstream of the TCR cassette. These cassettes were cloned into the pUC57 plasmid vector using the In-Fusion Snap Assembly Master Mix (Takara Bio Inc., Kusatsu, Shiga, Japan). Plasmid sequences were confirmed by Sanger sequencing. The entire construct was amplified from the plasmid using KOD One polymerase (Toyobo, Osaka, Japan) with M13 primers (Table S3), and the PCR product was purified using the NucleoSpin Gel and PCR Clean-up kit (Takara Bio Inc.) to be used as an HDR template.

### 2.8. CRISPR-Mediated Knock-In in Reporter T Cells

Cassettes encoding NY-ESO1-specific TCR (1G4) or LK117-derived TCRs with fluorescent barcodes were introduced into the reporter T cells via CRISPR/Cas9-mediated knock-in. In each well of a 24-well plate, 1 mL of culture medium was supplemented with 1.4 µL of Alt-R HDR Enhancer V2 (IDT) and prewarmed. Reporter T cells (4 × 10^5^) were resuspended in 20 µL of SE Cell Line Nucleofector Solution. A gRNA targeting the endogenous TCR β CDR3 region (CDR3β), 2 µg HDR template DNA for the NY-ESO1-specific TCR, and an equimolar pool (total 2 µg; 0.5 µg each) of four templates encoding distinct TCRs with unique fluorescent barcodes for multiplex knock-in were added. After a 2 min incubation, the mixture was electroporated using the 4D-Nucleofector System (program CL-120) and transferred to the prewarmed medium.

Knock-in Efficiency was assessed 72 h post-editing by flow cytometry as the percentages of CD3^+^ cells. Mean knock-in efficiency was 13.6% (range, 5–20%) for NY-ESO-1 specific TCRs and 6% (range, 5–6%) in LK117 TCR pools, and per-TCR compositions within CD3^+^ gate are summarized in Table S5. Prior to functional assays, CD3^+^ reporter T cells were purified on an SH800S cell sorter. For co-culture experiments with TMG-transfected EBV-LCLs, the barcode composition of the reporter pools was re-checked (Figure S5). We did not determine mono- versus bi-allelic editing frequencies in reporter T cells.

### 2.9. Whole-Exome Sequencing (WES)

DNA from tumor and matched normal tissues of the patient was subjected to WES. Sequencing reads were trimmed using trim-galore and aligned to the human reference genome (GRCh38) with BWA-MEM (v0.7.17) [23]. Somatic variants were called using Mutect2 (GATK v4.1.9) and Strelka [24], and germline variants were identified with HaplotypeCaller (GATK v4.1.9). The resulting variant calls were further annotated with ENSEMBL-VEP [25] and visually confirmed in Integrative Genomics Viewer (IGV, v2.19.5).

### 2.10. Bulk RNA-Seq

Total RNA from tumor tissue was analyzed by RNA-Seq. Reads were trimmed with trim-galore and mapped to GRCh38 using STAR (v2.7.8a) [26]. Gene expression levels were quantified with featureCounts (v1.6.4) [27], and fragments per kilobase of transcript per million mapped reads (FPKM) values were calculated in R. RNA-variant allele frequency (VAF) values were obtained by counting reference and variant-supporting reads at mutation sites using bam-readcount on RNA-Seq BAM files.

### 2.11. HLA Typing and Neoantigen Prediction

HLA class I alleles were inferred from WES data using OptiType [28]. Neoantigen candidates were predicted using pVACseq [29], integrating WES-derived somatic mutations with RNA expression data. Candidate peptides were filtered based on expression (FPKM > 10), VAF (RNA-VAF > 0.04) and predicted MHC binding affinity (IC_50_ < 500 nM, 9–10-mer peptides, MHCflurry [30] or NetMHCpan [31]).

### 2.12. Preparation of TMG Constructs and Transfection into EBV-LCLs

For each candidate missense mutation, we designed a DNA fragment encoding a 31–amino acid consisting of the mutated residue flanked by 15 amino acids on each side, which we designated a minigene. Five or six minigenes were concatenated in frame to generate TMG constructs without signal sequences or linker sequences, followed by T2A-eGFP for bicistronic expression. The corresponding DNA fragments were synthesized and cloned into the pCEP4 plasmid (Thermo Fisher Scientific) using In-Fusion Snap Assembly Master Mix according to the manufacturer’s instructions (Takara Bio Inc.).

Autologous EBV-LCLs (2–4 × 10^6^) were electroporated with the TMG plasmids using a 4D-Nucleofector X Unit with the SE Cell Line 4D-Nucleofector X Kit S (Lonza) and program DH-100. Immediately after electroporation, cells were incubated for 15 min at 37 °C, then transferred to prewarmed complete medium. At 24 h post-electroporation, hygromycin B (50 µg/mL) was added for selection and 2 days later, eGFP^+^ population was sorted on an SH800S cell sorter. The percentage of eGFP^+^ cells and eGFP mean fluorescence intensity (MFI) after sorting are shown in Figure S3.

### 2.13. Co-Culture of EBV-LCLs with Reporter T Cells

HLA-A*02:01–positive EBV-LCLs (1 × 10^5^) were resuspended in RPMI-1640 with 20% FBS and pulsed with NY-ESO-1 peptide (SLLMWITQV; 10^−5^–10^−12^ M, 10-fold serial dilutions) for 2 h at 37 °C, washed three times, and co-cultured overnight (12–16 h) with 1 × 10^5^ 1G4 TCR-expressing reporter T cells. For TMG-based screening and epitope mapping, reporter T cells carrying candidate TCR pools were co-cultured under identical conditions with LK117 EBV-LCLs expressing TMG constructs or with EBV-LCLs pulsed with the indicated synthetic peptides. After co-culture, supernatants were collected and IL-2 was quantified with IL-2 Human Uncoated ELISA Kit (Thermo Fisher Scientific). Cells were resuspended in medium containing 150 µg/mL luciferin (AAT Bioquest, Pleasanton, CA, USA) and bioluminescence was measured on a plate reader (TriStar LB941, Berthold Technologies GmbH & Co. KG, Bad Wildbad, Germany). Then the cells were stained with Pacific Blue anti-CD19 and PE anti-CD69 antibodies (BioLegend) and acquired on the CytoFLEX S flow cytometer (Beckman Coulter, Brea, CA, USA) for eGFP expression and CD69 upregulation on the CD19^−^ reporter T cells. Dose–response curves were fitted using a four-parameter logistic model in GraphPad Prism (v10.2.2, Sigmoidal, 4PL, X is concentration) to derive EC_50_ values.

For luciferase readouts, a positivity threshold was defined on each plate using on-plate negative controls: six wells containing reporter T cells co-cultured with untransfected, peptide-free EBV-LCLs. A response was called positive if its luminescence exceeded the plate-specific mean plus two standard deviations (mean + 2 SD) of these background wells. Each condition was assayed in one well per experiment (no technical replicates). The experiment was independently repeated twice. Given the small sample size, confidence intervals were not calculated.

### 2.14. Co-Culture of K562-Based Mono-Allelic HLA APCs with Reporter T Cells

HLA-B*54:01 and HLA-C*14:02 cDNAs were obtained from the RIKEN BioResource Research Center (Tsukuba, Japan). Each allele was subcloned into the pLenti-III-EF1α vector, and lentiviral particles were produced and concentrated as described above. K562 cells (JCRB Cell Bank, Osaka, Japan) were transduced and stained with APC anti-human HLA-A,B,C antibody; HLA-positive cells were isolated by cell sorting to generate mono-allelic K562 APC lines.

For co-culture assays, 1 × 10^5^ HLA-expressing K562 cells were pulsed with the indicated peptide at 1 μM for 2 h at 37 °C, then co-cultured overnight (12–16 h) with 1 × 10^5^ reporter T cells. Luciferase activity was quantified as described above.

## 3. Results

### 3.1. Establishment of the Jurkat-Based Reporter System

**Figure 1:**
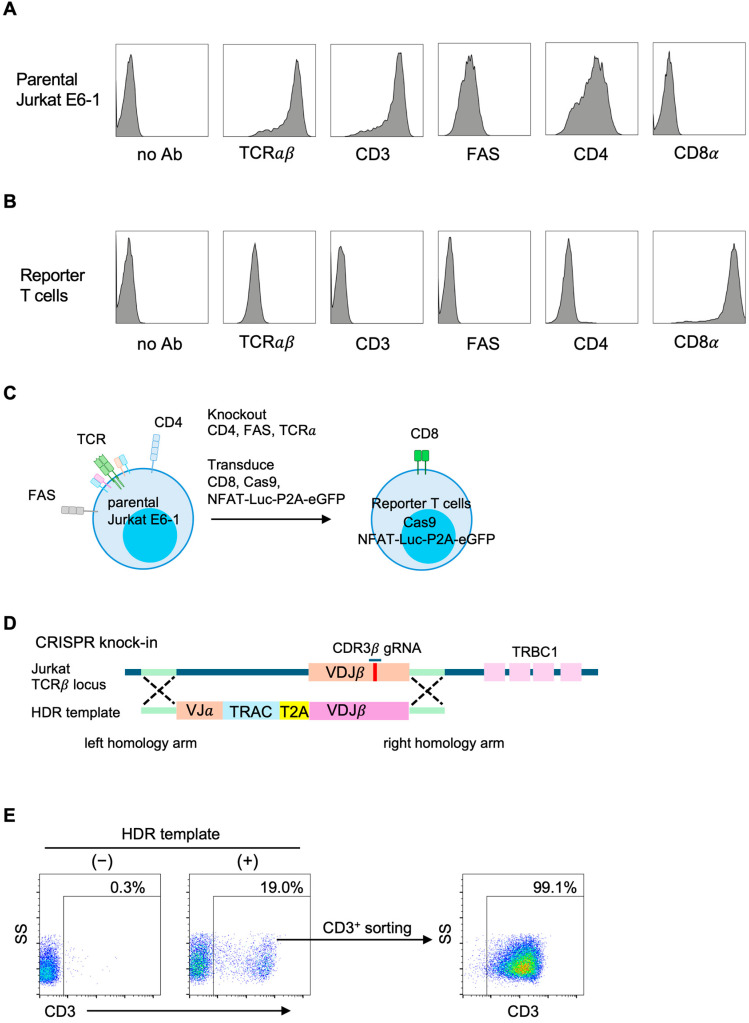
Establishment of reporter T cells and CRISPR/Cas9-mediated knock-in of a candidate T cell receptor (TCR). (**A**) Surface marker expression of parental Jurkat E6-1 cells. Histograms show the expression levels of the indicated markers. (**B**) Surface marker expression of engineered reporter T cells. Histograms show the expression levels of the indicated markers. (**C**) Schematic overview of the genetic modifications performed to generate reporter T cells. Parental Jurkat E6-1 cells were subjected to CRISPR/Cas9-mediated knockout of CD4, FAS, and TCRα, and lentiviral transduction of CD8α, CD8β, Cas9, and the NFAT–Luc–P2A–eGFP reporter cassette. (**D**) Targeted reconstitution of the TCR in reporter T cells at the endogenous TCRβ locus. (**E**) Flow cytometric analysis of CD3 restoration in reporter T cells after targeted reconstitution with the NY-ESO-1–specific 1G4 TCR. CD3^+^ cells were sorted to generate a uniform population of TCR-expressing reporter T cells. Pseudocolor plots indicate event density (blue, lower; red, higher).

To establish NeoPAIR-T, a multiplexed platform for the parallel identification of true neoantigens and their cognate TCRs, we developed a Jurkat-based reporter component using CRISPR/Cas9 engineering. We first characterized the parental Jurkat E6-1 cell line (ATCC, TIB-152) by flow cytometry. As shown in Figure 1A, Jurkat E6-1 cells expressed uniform levels of TCRαβ, CD3, FAS and CD4, but lacked CD8 expression.

Our reporter T cells were established based on the strategy reported by Vazquez-Lombardi et al. [18] with several modifications. Initially, CD4 and FAS were disrupted by Cas9 RNP electroporation, and CD4^−^FAS^−^ cells were sorted (Figure S1A). A key difference from the aforementioned study was lentiviral transduction for the stable expression of CD8 and Cas9 (Figure S1B), instead of CRISPR/Cas9-mediated knock-in, due to the higher efficiency of the lentiviral approach. Using this Cas9-transduced line, we performed TCRα knockout by electroporating a TRAC-targeting gRNA. CD3^−^ cells were then sorted, which indirectly enriched the Cas9^+^ population, as only Cas9-expressing cells underwent TRAC disruption. To enable quantitative monitoring of TCR signaling, we further introduced an NFAT response element-Luciferase-P2A-eGFP reporter cassette via lentiviral transduction (Figure S1B). After G418 selection, cells were stimulated with PMA and ionomycin, and eGFP^+^ cells were subsequently sorted, thus establishing the final reporter T cells (Figure 1B,C).

Candidate TCRs were subsequently introduced into the engineered reporter T cells by CRISPR/Cas9-mediated knock-in, ensuring physiological TCR expression and minimizing random integration. Specifically, the TCRα and TCRβ chains were linked via a T2A self-cleaving peptide and assembled into a HDR template targeted to the endogenous TCRβ locus (Figure 1D). The HDR template encoded the full-length TCRα chain (including VJ segments and the TRAC region) together with only the V(D)J segment of the TCRβ chain, relying on the endogenous TRBC1 constant region for proper expression. The gRNA used for targeting the TCRβ locus was designed within the CDR3 region and was Jurkat-specific, enabling precise single-allele editing.

As a model TCR, we first introduced an HLA-A*02:01–restricted NY-ESO-1–specific TCR (1G4) into the engineered reporter T cells. The mean knock-in efficiency was 13.6% (range, 5–20%), as determined by CD3 expression in flow cytometry, and CD3^+^ cells were subsequently enriched by sorting to generate a uniform population for downstream assays (Figure 1E).

Collectively, these modifications established a stable reporter T cell line suitable for the simultaneous screening of neoantigens and their cognate TCRs.

### 3.2. Functional Validation of the Reporter T Cells

**Figure 2:**
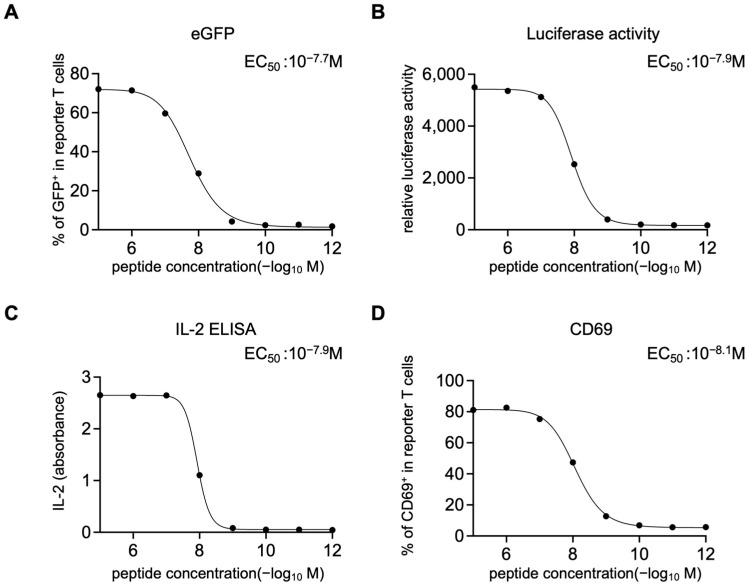
Functional validation of the reporter T cells. Reporter T cells expressing the 1G4 T cell receptor (TCR) were co-cultured overnight with HLA-A*02:01–positive Epstein–Barr virus-transformed lymphoblastoid cell lines (EBV-LCLs) that had been pulsed for two hours with NY-ESO-1 peptide at various concentrations and subsequently washed. (**A**) Frequency of eGFP-expressing cells in CD19^−^ reporter T cells analyzed by flow cytometry. (**B**) Luciferase activity measured after addition of D-luciferin. (**C**) IL-2 levels in culture supernatants determined by ELISA. (**D**) Frequency of CD69-expressing reporter T cells in CD19^−^ cells analyzed by flow cytometry. Each condition was measured in one well per experiment. Data shown are from one experiment, representative of two independent experiments.

To functionally validate the newly established reporter T cells, HLA-A*02:01-positive EBV-LCLs were pulsed with NY-ESO-1 peptide (SLLMWITQV) at various concentrations for two hours, washed and co-cultured with 1G4 TCR-knock-in reporter T cells overnight. Antigen-specific activation was clearly detected across multiple readouts: eGFP expression (Figure 2A), luciferase activity (Figure 2B), IL-2 secretion (Figure 2C) and CD69 upregulation (Figure 2D). Importantly, all four readouts exhibited minimal background signal in the absence of peptide stimulation.

The dose–response curves obtained from eGFP, luciferase, IL-2, and CD69 readouts showed similar profiles, with EC_50_ values ranging from 10^−8.1^ to 10^−7.7^ M (Figure 2A–D).

Collectively, these data demonstrate that the engineered reporter T cells faithfully recapitulate antigen-specific TCR signaling with multiple quantitative readouts, validating its utility as a robust platform for subsequent screening of candidate TCRs.

### 3.3. Overview of the NeoPAIR-T Workflow

**Figure 3:**
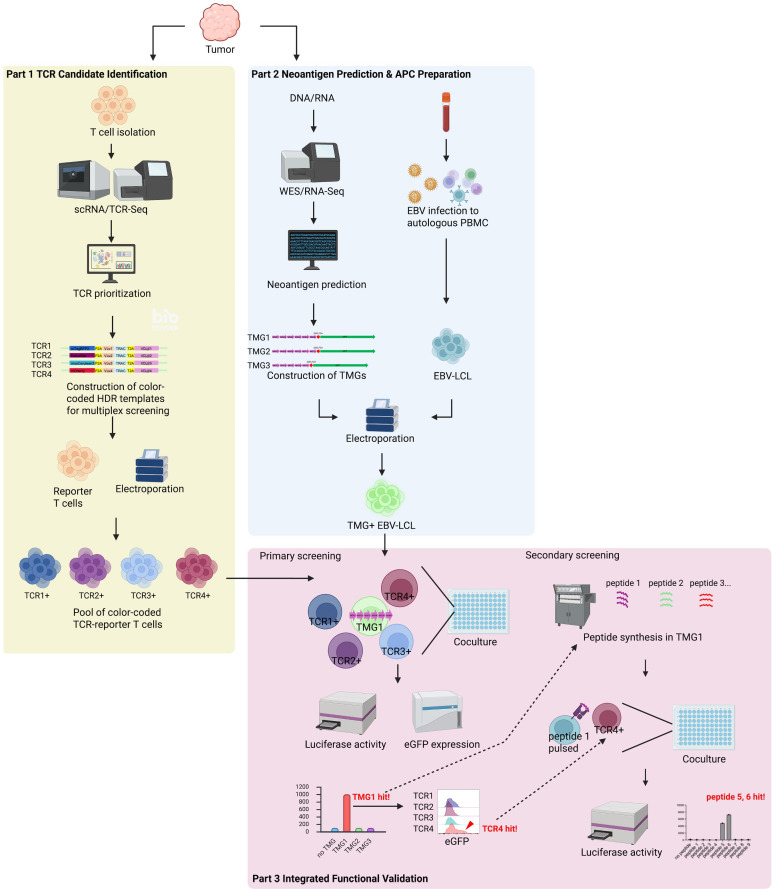
Schematic overview of the integrated three-part workflow of the NeoPAIR-T. The figure illustrates the NeoPAIR-T workflow applied to the LK117 clinical sample, which is conceptually divided into three interconnected and sequential parts. **Part 1** (T cell receptor (TCR) Candidate Identification): Tumor-infiltrating lymphocytes are isolated from the tumor for single-cell RNA and TCR sequencing. Candidate TCRs are prioritized based on transcriptomic features (e.g., CXCL13 expression, NeoTCR8 score) and clonality. Homology-directed repair (HDR) templates for each prioritized TCR, barcoded with a unique fluorescent protein (e.g., mCherry, mTagBFP2), are constructed and electroporated into the engineered reporter T cells to generate a multiplexed pool of color-coded TCR–reporter T cells. **Part 2** (Neoantigen Prediction & APC Preparation): In parallel, DNA and RNA are extracted from the tumor tissue for whole-exome and RNA sequencing to predict neoantigen candidates. From the patient’s peripheral blood, autologous Epstein–Barr virus-transformed lymphoblastoid cell lines (EBV-LCLs) are established to serve as antigen-presenting cells (APCs). The predicted neoantigen candidates are synthesized as tandem minigene (TMG) constructs and electroporated into the autologous EBV-LCLs. **Part 3** (Integrated Functional Validation): This validation phase consists of two sequential steps. In the primary screening, the TMG-expressing EBV-LCLs are co-cultured with the multiplexed TCR–reporter T cell pool. A ‘hit’ is identified when a specific TMG elicits a bulk luciferase signal. The cognate TCR is then deconvoluted by identifying the color-coded reporter subset (e.g., mCherry^+^ TCR4) that upregulates the eGFP reporter, as determined by flow cytometry. In the secondary screening, individual peptides encoded within the ‘hit’ TMG (e.g., TMG1) are synthesized. These peptides are then pulsed onto EBV-LCLs and co-cultured with the corresponding ‘hit’ TCR–reporter T cells (e.g., TCR4^+^) to identify the minimal epitope responsible for reporter activation, as assessed by luciferase activity. Created in BioRender. Nagaoka, K. (2025) https://BioRender.com/m537tcc (accessed on 12 November 2025).

With the reporter T cells’ performance confirmed using a model antigen, we next applied NeoPAIR-T to a clinical sample from a patient with lung squamous cell carcinoma (LK117). In Figure 3, we show an overview of the three-part workflow: first, the identification of candidate tumor-reactive TCRs via single-cell sequencing; second, the parallel prediction of neoantigen candidates from the same tumor’s genomic and transcriptomic data; and finally, the functional validation of the resulting candidate pairs using our integrated platform. The following sections describe the execution of this workflow, beginning with the identification of candidate TCRs.

### 3.4. Selection of Candidate TCRs from Single-Cell RNA/TCR-Seq Data of a Lung Squamous Cell Carcinoma Patient

We sorted CD8^+^ TILs from the LK117 tumor (Figure S2) and performed single-cell RNA/TCR-Seq. Gene expression and paired TCRαβ information were obtained from 4476 cells. This analysis revealed multiple expanded clonotypes within the tumor-infiltrating CD8^+^ T cell compartment. First, we selected the five most expanded clonotypes (534, 232, 169, 101, and 92 cells, respectively; Table S4) among tumor-infiltrating CD8^+^ T cells as initial candidates, since highly frequent clonotypes are often enriched for neoantigen-specific T cells in lung cancers [32].

**Figure 4:**
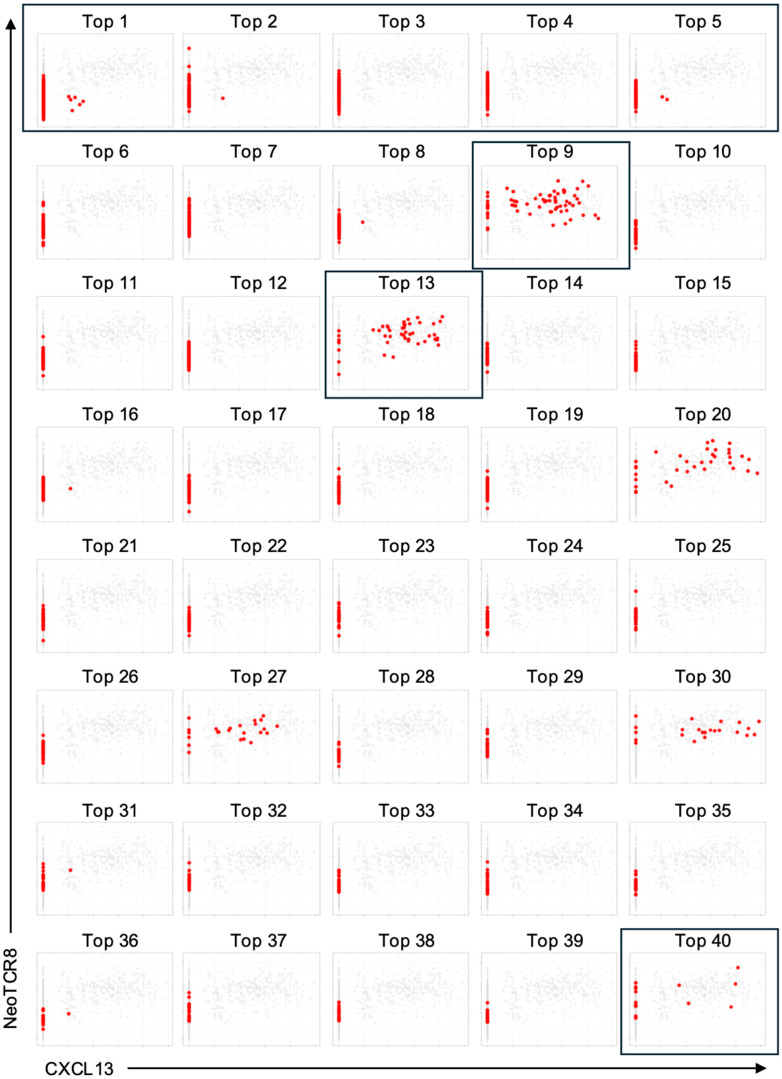
Candidate T cell receptor (TCR) selection from single-cell RNA/TCR-sequencing data of LK117, a lung cancer patient. Single-cell RNA/TCR sequencing data from CD8^+^ T cells of the LK117 tumor sample were analyzed to calculate neoTCR8 scores using single-sample gene set enrichment analysis (ssGSEA). The figure displays all CD8^+^ T cells as gray dots, representing the complete dataset shared across all panels. Each panel corresponds to one clonotype, with red dots indicating individual T cells assigned to that clonotype, plotted according to their neoTCR8 score and CXCL13 expression. Candidate TCRs selected for reconstruction included the top 1–5 clonotypes by frequency as well as clonotypes 9, 13, and 40, which exhibited both high neoTCR8 scores and CXCL13 expression (highlighted with boxes). Single-cell RNA/TCR sequencing was performed once.

To further enrich for tumor-reactive candidates, we next examined the expression of CXCL13 and the NeoTCR8 score, a transcriptomic signature of neoantigen-reactive CD8^+^ T cells [13,33,34,35,36]. From the top 40 clonotypes by frequency, we selected three additional clonotypes showing high CXCL13 expression and elevated NeoTCR8 scores (Figure 4).

In total, eight clonotypes (five expanded and three CXCL13^+^) were reconstructed as CRISPR/Cas9 knock-in HDR templates (Table S5).

### 3.5. Multiplex Knock-In of LK117-Derived Candidate TCRs into Reporter T Cells

**Figure 5:**
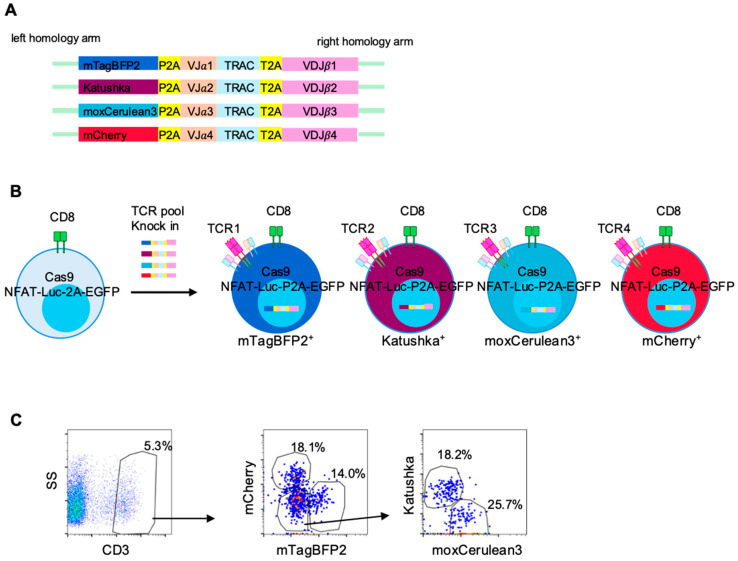
One-step multiplex knock-in of T cell receptors (TCRs) into reporter T cells. (**A**) Knock-in constructs encoding four TCRs, each linked to a distinct fluorescent protein (mTagBFP2, Katushka, moxCerulean3 or mCherry). (**B**) Schematic overview of the multiplex TCR knock-in system. The four constructs were pooled and introduced into reporter T cells. Cells expressing different fluorescent proteins correspond to distinct TCRs. (**C**) Three days after electroporation, CD3 expression in reporter T cells was analyzed by flow cytometry. Expression of mCherry, mTagBFP2, Katushka, and moxCerulean3 within the CD3^+^ population is shown, with numbers indicating the percentage of each fluorescent subset among CD3^+^ cells. Pseudocolor plots indicate event density (blue, lower; red, higher).

To enable parallel functional analysis of the selected LK117-derived TCRs, we constructed four distinct HDR donor templates, each encoding a different candidate TCR linked via a P2A sequence to a unique fluorescent reporter (mCherry, mTagBFP2, Katushka, or moxCerulean3) (Figure 5A). The four HDR templates were then pooled and simultaneously introduced into the engineered reporter T cells in a single CRISPR/Cas9-mediated knock-in reaction targeting the endogenous TCRβ locus (Figure 5B). This one-step multiplex knock-in successfully generated four distinct reporter T cell populations, each expressing one of the four fluorescent markers, as confirmed by flow cytometric analysis (Figure 5C). Importantly, individual cells exhibited only a single fluorescent color, indicating that each cell incorporated one TCR construct exclusively. The respective knock-in efficiencies were 18.1% for mCherry, 14.0% for mTagBFP2, 18.2% for Katushka, and 25.7% for moxCerulean3, confirming that multiple TCRs can be introduced and tracked in parallel under identical editing conditions (Figure 5C). These results demonstrate the feasibility of multiplex knock-in using fluorescent barcoding, enabling parallel functional evaluation of multiple candidate TCRs within a single experimental system.

### 3.6. Prediction and Construction of Neoantigen TMGs for EBV-LCL Presentation

**Figure 6:**
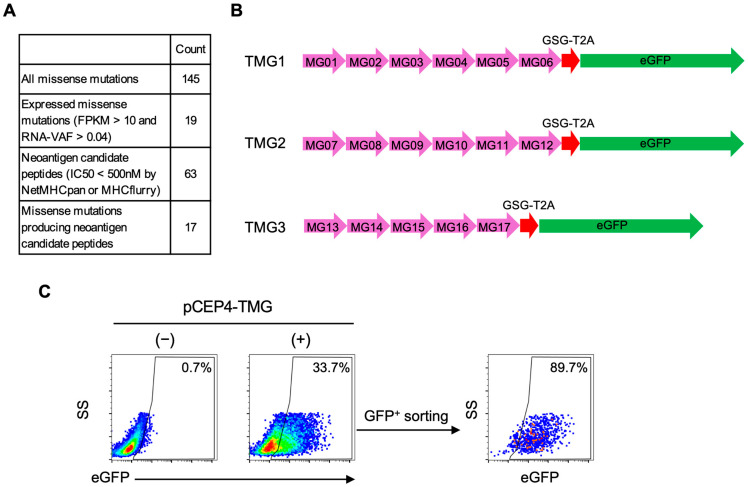
Design and construction of neoantigen tandem minigenes (TMGs) for expression in Epstein–Barr virus-transformed lymphoblastoid cell lines (EBV-LCLs). (**A**) Whole-exome sequencing (WES) and RNA sequencing (RNA-Seq) data from the LK117 tumor were processed for in silico neoantigen prediction. From 145 missense mutations, 19 were expressed (FPKM > 10 and RNA variant allele frequency (RNA-VAF) > 0.04). HLA class I binding prediction (NetMHCpan/MHCflurry; IC_50_ < 500 nM) for 9- or 10-mer peptides yielded 63 candidate peptides arising from 17 missense mutations. (**B**) Seventeen minigenes encoding these mutations were designed and assembled into three TMG constructs, which were cloned into an EBV episomal expression vector. (**C**) The TMG constructs were introduced into autologous LK117 EBV-LCLs by electroporation. eGFP^+^ cells were sorted to enrich TMG-expressing populations. Pseudocolor plots indicate event density (blue, lower; red, higher).

While establishing the reporter T cells, we executed the second part of NeoPAIR-T workflow: the prediction of neoantigen candidates and the preparation of autologous APCs (Figure 3). To predict potential neoantigens from the LK117 lung squamous cell carcinoma sample, we first extracted DNA and RNA from tumor tissue and performed WES and bulk RNA-Seq. WES identified 145 missense mutations (Figure 6A, Table S6). Concurrently, HLA typing was performed and revealed HLA-A*24:02, HLA-A*31:01, HLA-B*51:01, HLA-B*54:01, HLA-C*01:02, and HLA-C*14:02 in this patient.

Using these data, we performed MHC class I binding prediction for somatic missense mutations. Candidate epitopes were selected based on tumor RNA expression (FPKM > 10), VAF (RNA-VAF > 0.04), and predicted binding affinity (IC_50_ < 500 nM for 9- or 10-mer peptides calculated by MHCflurry or NetMHCpan). This filtering yielded 94 predicted candidate epitopes derived from 17 distinct mutated genes (Table S7). As some peptides were predicted to bind to multiple HLA alleles of the patient, removal of these duplicates resulted in 63 unique peptide candidates (Table S8). To efficiently screen this large number of candidates without laborious individual peptide synthesis, we concatenated the selected mutations and assembled them into three TMGs encoding the predicted neoantigen repertoire (Figure 6B, Table S9) [37].

Each minigene was designed as a 31-amino-acid sequence, centered on the mutated residue and flanked by 15 amino acids of the corresponding wild-type sequence; in total, we designed 17 minigenes, designated MG01–MG17 (Table S9). Each TMG construct consisted of five to six such minigenes, collectively encoding multiple predicted neoantigen peptides. A single minigene could generate several candidate peptides differing in length (9- or 10-mers), mutation position, or predicted HLA restriction. For example, MG01 encoded one peptide, whereas MG07 and MG08 within TMG2 encoded six and seven distinct peptides, respectively (Figure S4). Consequently, TMG1, TMG2, and TMG3 encoded 9, 38, and 16 neoantigen candidate peptides, respectively (Figure S4 and Table S8). These TMGs were cloned into an EBV-episomal expression vector (pCEP4) and introduced into autologous EBV-LCLs by electroporation. After transfection, eGFP^+^ cells were sorted to enrich TMG-expressing cells (Figure 6C). Thus, by integrating neoantigen prediction, TMG construction, and EBV-LCL transfection, we established an autologous APC platform for screening candidate TCRs against the patient’s neoantigen repertoire.

### 3.7. Screening of TCR–Neoantigen Pairs

As the final step of the workflow, we proceeded to Part 3: the integrated functional validation of the candidate pairs (Figure 3). To functionally identify cognate TCR–neoantigen pairs, we introduced two pools of candidate TCRs (Pool 1: TCR1–4; Pool 2: TCR5–8) into engineered reporter T cells and co-cultured them with autologous EBV-LCLs expressing TMG1-3. In total, six co-culture combinations (Pool 1 × TMG1–3 and Pool 2 × TMG1–3) were tested, enabling functional screening of eight TCRs against 63 predicted neoepitopes encoded by 17 minigenes.

**Figure 7:**
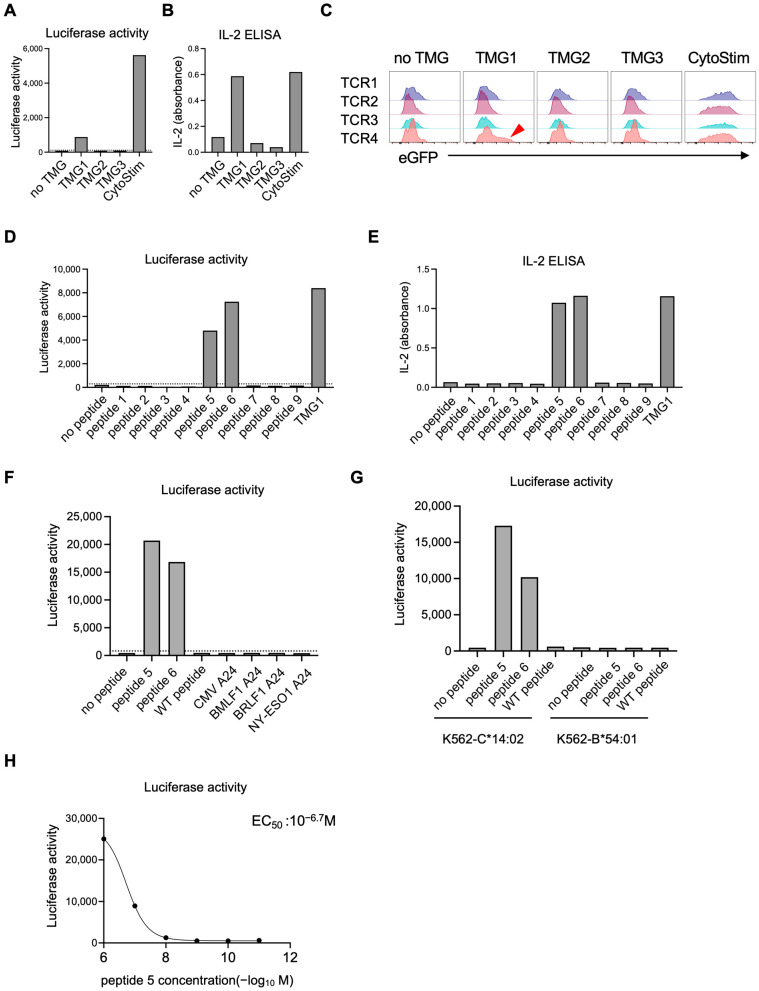
Screening of T cell receptor (TCR)–neoantigen pairs (Pool 1). Eight candidate TCRs were divided into two pools (Pool 1 and Pool 2), each containing four TCRs tagged with distinct fluorescent proteins to enable their discrimination. Each TCR was first constructed as an individual knock-in template. For multiplex knock-in, the four templates within Pool 1 were combined and simultaneously introduced into reporter T cells using CRISPR/Cas9-mediated knock-in. After sorting for CD3^+^ cells, which confirmed successful knock-in, the resulting reporter T cell pools expressing four color-coded TCRs were co-cultured with Epstein–Barr virus-transformed lymphoblastoid cell lines (EBV-LCLs) transfected with tandem minigenes (TMGs) for parallel functional screening. (**A**,**B**) Reporter T cells carrying Pool 1 TCRs responded selectively to TMG1-expressing EBV-LCLs, as measured by luciferase activity (**A**) and IL-2 secretion (**B**). (**C**) Flow cytometric analysis revealed that the mCherry^+^ subset (TCR4) specifically upregulated eGFP in response to TMG1 (arrowhead). (**D**,**E**) mCherry^+^ TCR4-expressing reporter T cells were isolated and co-cultured with EBV-LCLs pulsed with synthetic peptides derived from TMG1 (peptides 1–9). Luciferase assays (**D**) and IL-2 ELISA (**E**) identified peptide-specific activation. (**F**) mCherry^+^ TCR4-expressing reporter T cells were co-cultured with EBV-LCLs pulsed with peptide 5, peptide 6, the corresponding wild-type (WT) peptide or irrelevant peptides. Luciferase assay revealed specificity of TCR to mutated peptides. (**G**) mCherry^+^ TCR4-expressing reporter T cells were co-cultured with K562 expressing HLA-C*14:02 or HLA-B*54:01 and pulsed with indicated peptides. Luciferase assay indicated restriction by HLA-C*14:02. (**H**) mCherry^+^ TCR4-expressing reporter T cells were co-cultured with EBV-LCLs that had been pulsed for two hours with peptide 5 at various concentrations. Luciferase activity was measured to generate a dose–response curve. Each condition was measured in one well per experiment. Data shown are from one experiment, representative of two independent experiments. The dashed horizontal line indicates the mean + 2 SD calculated from six on-plate negative-control wells.

In the first screening (Pool 1), luciferase assays and IL-2 ELISA revealed that reporter T cells carrying Pool 1 TCRs responded selectively to TMG1-expressing EBV-LCLs (Figure 7A,B). Flow-cytometric analysis of the multiplexed population identified the mCherry^+^ subset (TCR4) as the reactive clone, showing eGFP upregulation upon stimulation (Figure 7C and Figure S5). To map the precise antigen, we synthesized nine peptides (peptides 1–9) corresponding to mutations contained in TMG1 (Figure S4 and Table S8). Co-culture assays demonstrated that peptide 5 (NYICNSSCM) and peptide 6 (YNYICNSSCM) both activated TCR4-reporter T cells (Figure 7D,E). These peptides represented length variants derived from the same mutant TP53 M237I missense mutation, confirming that TP53 M237I was a neoantigen and that TCR4 was its cognate receptor. TCR4-reporter T cells responded to mutated peptides, but not to the corresponding wild-type peptide or irrelevant control peptides (Figure 7F). Using K562-based APCs expressing a single HLA allele, we identified HLA-C*14:02 as the restricting allele (Figure 7G). Dose–response titrations were performed by pulsing EBV-LCLs with peptide 5 at graded concentrations. Dose–response curves yielded an EC_50_ of 10^−6.7^ M (Figure 7H).

**Figure 8:**
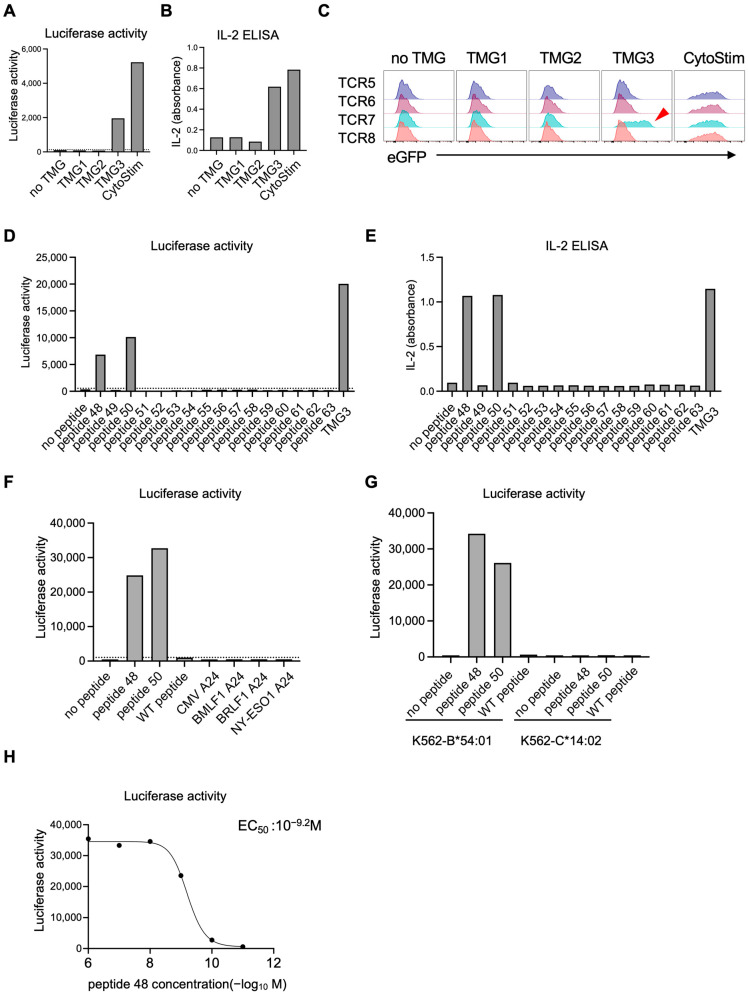
Screening of T cell receptor (TCR)–neoantigen pairs (Pool 2). Eight candidate TCRs were divided into two pools (Pool 1 and Pool 2), each containing four TCRs tagged with distinct fluorescent proteins to enable their discrimination. Each TCR was first constructed as an individual knock-in template. For multiplex knock-in, the four templates within Pool 2 were combined and simultaneously introduced into reporter T cells using CRISPR/Cas9-mediated knock-in. After sorting for CD3^+^ cells, which confirmed successful knock-in, the resulting reporter T cell pools expressing four color-coded TCRs were co-cultured with Epstein–Barr virus-transformed lymphoblastoid cell lines (EBV-LCLs) transfected with tandem minigenes (TMGs) for parallel functional screening. (**A**,**B**) Reporter T cells carrying Pool 2 TCRs responded selectively to TMG3-expressing EBV-LCLs, as measured by luciferase activity (**A**) and IL-2 secretion (**B**). (**C**) Flow cytometric analysis revealed that the moxCerulean3^+^ subset (TCR7) specifically upregulated eGFP in response to TMG3 (arrowhead). (**D**,**E**) moxCerulean3^+^ TCR7-expressing reporter T cells were isolated and co-cultured with EBV-LCLs pulsed with synthetic peptides derived from TMG3 (peptides 48–63). Luciferase assays (**D**) and IL-2 ELISA (**E**) identified peptide-specific activation. (**F**) moxCerulean3^+^ TCR7-expressing reporter T cells were co-cultured with EBV-LCLs pulsed with peptide 48, peptide 50, the corresponding wild-type (WT) peptide or irrelevant peptides. Luciferase assay revealed specificity of TCR to mutated peptides. (**G**) moxCerulean3^+^ TCR7-expressing reporter T cells were co-cultured with K562 expressing HLA-B*54:01 or HLA-C*14:02 and pulsed with indicated peptides. Luciferase assay indicated restriction by HLA-B*54:01. (**H**) moxCerulean3^+^ TCR7-expressing reporter T cells were co-cultured with EBV-LCLs that had been pulsed for two hours with peptide 48 at various concentrations. Luciferase activity was measured to generate a dose–response curve. Each condition was measured in one well per experiment. Data shown are from one experiment, representative of two independent experiments. The dashed horizontal line indicates the mean + 2 SD calculated from six on-plate negative-control wells.

In a parallel experiment (Pool 2), reporter T cells carrying Pool 2 TCRs responded to TMG3-expressing EBV-LCLs (Figure 8A,B). Within this pool, flow-cytometric analysis identified the moxCerulean3^+^ subset (TCR7) as the responding clone, marked by eGFP induction (Figure 8C and Figure S5). To determine the precise epitope, we synthesized 16 peptides (peptides 48–63) derived from TMG3 (Figure S4 and Table S8). Functional assays revealed that peptide 48 (EAMALGLEA) and peptide 50 (EAMALGLEAA) triggered robust activation of TCR7-reporter T cells (Figure 8D,E). Both epitopes were length variants derived from the same mutant POLD1 R776L missense mutation, confirming that POLD1 R776L was a neoantigen and that TCR7 was its cognate receptor. TCR7-reporter T cells responded to mutated peptides, but not to the corresponding wild-type peptide or irrelevant control peptides (Figure 8F). Using K562-based APCs expressing a single HLA allele, we identified HLA-B*54:01 as the restricting allele (Figure 8G). Dose–response curves yielded an EC_50_ of 10^−9.2^ M (Figure 8H).

Together, these experiments demonstrate that the NeoPAIR-T system enables stepwise identification of cognate TCR–neoantigen pairs, from pooled multiplex screening to precise mapping of minimal epitopes at the peptide level.

## 4. Discussion

In this study, we developed and validated NeoPAIR-T, a novel, integrated platform designed to overcome a major bottleneck in neoantigen-based immunotherapy—the functional identification of authentic tumor-specific neoantigens recognized by cognate TCRs. NeoPAIR-T, the overall workflow of which is illustrated in Figure 3, enables multiplexed identification of bona fide neoantigen–TCR pairs by systematically integrating transcriptome-guided prediction of tumor-specific TCRs with MHC-binding–based prediction of candidate neoantigens, their efficient expression in autologous APCs, and functional screening using reporter T cells. This approach allows the discrimination of true neoantigen-specific T cells from bystander T-cell noise and the experimental confirmation of authentic neoantigen–TCR interactions among numerous in silico predicted candidates.

A primary challenge in functional validation of neoantigen–TCR interactions arises from the low accuracy of in silico prediction of neoantigen peptides, which generates a vast number of false-positive candidates. Indeed, a meta-analysis of 13 studies reported that fewer than 2.7% of neopeptides prioritized by bioinformatics pipelines were actually recognized by patient-derived T cells [9,38]. Similarly, a large-scale benchmarking study by the Tumor Neoantigen Selection Alliance (TESLA) consortium demonstrated a success rate of only about 6% [39]. Consistent with these reports, in our screen 4 of 63 predicted peptides (6.3%) elicited measurable TCR-dependent activation, reinforcing that current prediction algorithms remain insufficiently precise. This consistently low predictive accuracy necessitates the synthesis and testing of hundreds to thousands of candidate peptides, thereby making a robust and efficient experimental validation process indispensable for distinguishing the few truly immunogenic neoantigens from the many non-functional predictions [16,40]. To address this limitation, we began with 145 mutated genes in LK117, from which in silico prediction identified 63 candidate peptides derived from 17 missense mutations. Minigenes from the 17 missense mutations were concatenated into three TMG constructs for primary screening. Whereas a peptide-synthesis-dependent approach would require synthesizing all 63 candidate peptides, epitope mapping in our workflow required synthesis of only 25 peptides (9 from TMG1 and 16 from TMG3, Figure S4), thereby improving overall efficiency. To further improve accuracy, future work will incorporate presentation-aware prioritization (e.g., proteasomal cleavage, TAP transport and peptide–HLA complex stability), and where available, cross-checks with immunopeptidomics to mitigate EBV-LCL presentation bias. At the same time, these intrinsically low hit-rates highlight the necessity of functional screening platforms capable of testing large candidate sets efficiently. The NeoPAIR-T system described here directly addresses this methodological gap by providing an efficient and versatile workflow for pairing neoantigens with cognate TCRs, thereby overcoming one of the central bottlenecks in neoantigen discovery.

In the LK117 case, a standard peptide-synthesis-dependent assay would require 8 TCRs × 63 peptides = 504 wells. By contrast, NeoPAIR-T condensed the screen to 2 pools × 3 TMGs co-cultures (6 wells), followed by peptide-level deconvolution for the two hit TCRs (9 and 16 wells), totaling 31 wells—an approximately 94% reduction—while delivering two quantitative readouts (luciferase and eGFP) from the same wells.

Our platform also addresses another critical set of challenges in functional validation: the reliability and throughput of TCR assessment. Traditional TCR transduction methods using retroviral or lentiviral vectors suffer from two inherent problems. The first is mispairing with endogenous TCR chains, which poses a risk of unpredictable off-target reactivities [41,42]. The second is random genomic integration, which, being a process governed by Poisson statistics, inevitably generates a heterogeneous cell population with variable transgene copy numbers [43]. This inherent heterogeneity leads to unpredictable functional variability and precludes clean, multiplexed screening from a pooled library. To mitigate these issues, we combine TCRα-knockout with a targeted knock-in into the endogenous TCRβ CDR3 region. Using this approach, we established NY-ESO-1-specific 1G4 TCR knock-in reporter T cells, which showed luciferase and eGFP induction, CD69 upregulation and IL-2 secretion upon stimulation with the HLA-A*02:01-restricted NY-ESO1 peptide, with EC_50_ values similar to those reported previously [18]. This approach not only ensures physiological, mispairing-free expression but also guarantees monoallelic, single-copy integration. This “one-cell, one-TCR” homogeneity is fundamental to our multiplexing strategy, making it possible to barcode each TCR with a distinct fluorescent protein and screen multiple candidates in parallel, thereby drastically accelerating the validation workflow. In practice, this configuration enables functional screening of four distinct TCR–T cell clones against a single antigen-presenting cell expressing approximately ten candidate neoantigens, allowing efficient identification of matched neoantigen–TCR pairs with minimal experimental complexity.

In this study, we prioritized TCR candidates based on two key features associated with tumor reactivity: high clonal expansion and transcriptomic signatures such as CXCL13 expression and the NeoTCR8 signature score. Functional validation of two neoantigen-reactive TCRs (TCR4 and TCR7) selected through this data-driven approach using the NeoPAIR-T platform provides proof of concept for the feasibility and robustness of our integrated workflow. However, this validation was performed in a single case, and the optimal criteria for prioritizing TCRs remains an area of active investigation. While our results support the utility of transcriptomic markers, other studies, particularly in lung cancer, have highlighted the importance of high clonal frequency in addition to CD39 protein and CXCL13 mRNA expressions in tumor-reactive T cells [32]. Therefore, further studies across multiple patients and cancer types are necessary to delineate the relative importance and predictive value of transcriptomic signatures versus clonal expansion for prioritizing TCR candidates.

Of the eight candidate TCRs evaluated, only two were confirmed to recognize cognate neoantigens. While a fair evaluation requires comparison to an appropriate baseline (e.g., random TCR sampling), the limited success rate is consistent with previous reports that the majority of tumor-infiltrating lymphocytes are bystander T cells lacking tumor specificity [11]. However, this outcome is also intrinsically linked to the design of our study, in which our neoantigen search was restricted to missense mutations. Indeed, other classes of somatic alterations and aberrant expression events—such as frameshift mutations, gene fusions, alternative splicing events, intron retention, translation from non-canonical open reading frames, and the expression of endogenous retroelements—are known to be important sources of neoantigens [40].

This particular limitation highlights a key future strength of our platform: its flexibility. The EBV-LCL-based TMG system readily accommodates these diverse mutation types into TMG constructs for evaluation within the same experimental framework. For example, we will encode the mutated open reading frame from the variant codon through to the first in-frame stop codon for frame-shift mutation. For gene fusions, if donor and acceptor genes are in-frame, a junction-spanning minigene centered on the breakpoint can be used; if the fusion introduces a frameshift, we will encode from the breakpoint to the first in-frame stop codon, analogous to the frameshift strategy. By expanding our search to include these variants in future studies, we anticipate the identification of broader and previously overlooked neoantigens and their cognate TCRs.

From a NeoPAIR-T-validated epitope–TCR pair, off-target risk should be reduced using complementary approaches, such as alanine scanning and cross-reactivity assessment against normal-cell panels. In parallel, manufacturability should be considered—either engineering TCR-T in autologous primary T cells or selecting and preparing appropriate vaccine modalities—as depicted by the intended translational path.

This study has several limitations. First, experimental validation was conducted in a single patient, which constrains generalizability; multi-patient and multi-cancer-type cohorts will be required to fully establish robustness, reproducibility, and clinical applicability. At the same time, the integrated platform developed here provides a foundation for such future work. By combining four spectrally distinct TCR reporter cell lines with autologous B-cell APCs that present peptides on the patient’s complete HLA repertoire and are engineered to express EBV-vector-based minigenes for antigen delivery, we created a flexible system for neoantigen interrogation. This architecture enables parallel, high-resolution functional assessment of diverse neoantigens and is expected to serve as a broadly useful discovery engine as we expand NeoPAIR-T to additional patients and cancer types. Second, although EBV-LCLs provide a practical and widely used APC platform for in vitro antigen presentation and enabled efficient screening in this study, EBV transformation may modify certain cellular characteristics, and differences from autologous tumor cells cannot be fully excluded. Therefore, validation using patient-derived tumor cells or tumor organoids will ultimately be required to confirm antigen presentation and functional recognition in a physiologically relevant setting. Such experiments were not feasible here due to limited availability of tumor material. Third, although the use of Jurkat-derived reporter cells enabled efficient identification of neoantigen-reactive TCRs, the present study did not include downstream validation using primary human T cells engineered with the identified TCRs. Such follow-up experiments, including assessment of cytotoxicity or cytokine responses against autologous tumor cell lines or patient-derived organoids, will be essential steps for advancing toward cancer vaccine development or TCR–T cell therapy. The current study addresses the step immediately before that stage: resolving one of the major bottlenecks in neoantigen immunology by establishing an efficient and versatile workflow for pairing neoantigens with their cognate TCRs. Our goal was to build a robust discovery engine, and future studies using primary T cells and patient-derived tumor materials will further extend the translational applicability of NeoPAIR-T. Fourth, we did not perform a formal passage-stability study of the Jurkat reporter line (e.g., longitudinal assessment of reporter responsiveness, phenotype, or knock-in retention across passages). Locus-specific copy/allelic assessment, per-locus indel quantification, experimental off-target profiling, and RT-qPCR of TMG transcripts were also not performed. On-target integration was inferred functionally from CD3 reconstitution, and TMG expression was monitored phenotypically by eGFP expression. Finally, because each TMG encodes multiple minigenes, intra-TMG competition for MHC class I loading may underrepresent low-affinity yet biologically relevant epitopes, resulting in potential false negatives. Although we diversified predicted HLA restrictions within each construct, competition-related false negatives cannot be excluded.

## 5. Conclusions

We have established an integrated platform that bridges the critical gap between candidate identification and functional validation in neoantigen research. By uniquely combining physiological TCR expression, simplified antigen presentation, and multiplexed screening, our platform provides a practical solution for the rapid discovery of functional TCR–neoantigen pairs. This work lays the groundwork for the efficient identification of functional TCR–neoantigen pairs, poised to accelerate the development of personalized neoantigen-based immunotherapies.

## Data Availability

All data generated or analyzed during this study are included in this published article and its Supplementary Information Files. Additional datasets generated and/or analyzed during the current study are available from the corresponding author upon reasonable request.

## Supplementary Materials

The following supporting information can be downloaded at: https://www.mdpi.com/article/10.3390/cells14221789/s1, Figure S1: Gene editing targets and lentiviral constructs for engineering reporter T cells; Figure S2: Gating strategy for CD8^+^ tumor infiltrating T cell sorting from LK117 tumor; Figure S3: eGFP expression in TMG-transfected EBV-LCLs after sorting; Figure S4: Tandem minigene (TMG) design and coverage of candidate HLA class I-binding peptides; Figure S5: Frequencies of individual reporter T cell populations in pooled cells; Table S1: Reagents used in this study; Table S2: crRNA sequences used in this study; Table S3: Primer sequences used in this study; Table S4: The 40 most frequent T cell receptor (TCR) clonotypes identified in tumor-infiltrating CD8^+^ T cells from the LK117 tumor; Table S5: Candidate T cell receptors (TCRs) from LK117 tumor; Table S6: Somatic missense mutations in LK117 tumor; Table S7: Predicted neoantigen peptide–HLA pairs from LK117; Table S8: Candidate neoantigen peptides from LK117; Table S9: Candidate minigenes from LK117.

## Abbreviations

The following abbreviations are used in this manuscript:

TCR	T cell receptor
APC	Antigen-presenting cell
TMG	tandem minigene
TCR-T	TCR-engineered T cell
PBMC	peripheral blood mononuclear cell
TIL	Tumor-infiltrating lymphocyte
EBV	Epstein–Barr virus
FBS	fetal bovine serum
EBV-LCL	Epstein–Barr virus-transformed lymphoblastoid cell line
gRNA	guide RNA
RNP	ribonucleoprotein
IDT	Integrated DNA Technologies
crRNA	CRISPR RNA
tracrRNA	trans-activating crRNA
TRAC	T cell receptor alpha constant
RNA/TCR-Seq	RNA/TCR sequencing
RNA-Seq	RNA sequencing
UMI	unique molecular identifier
ssGSEA	single-sample gene set enrichment analysis
HDR	homology-directed repair
WES	whole-exome sequencing
IGV	Integrative Genomics Viewer
VAF	variant allele frequency
FPKM	fragments per kilobase of transcript per million mapped reads
TESLA	Tumor Neoantigen Selection Alliance
