# Supplementary material for: NeoPAIR-T: Functional Mapping of Neoantigen–TCR Pairs Using a CRISPR-Engineered Jurkat Reporter System
Source: Cells. 2025 Nov 14;14(22):1789. doi: 10.3390/cells14221789 (PMC12650950; doi:10.3390/cells14221789)
Supplement: Supplementary file 1 [file cells-14-01789-s001.zip › Supplementary Figures.pdf]

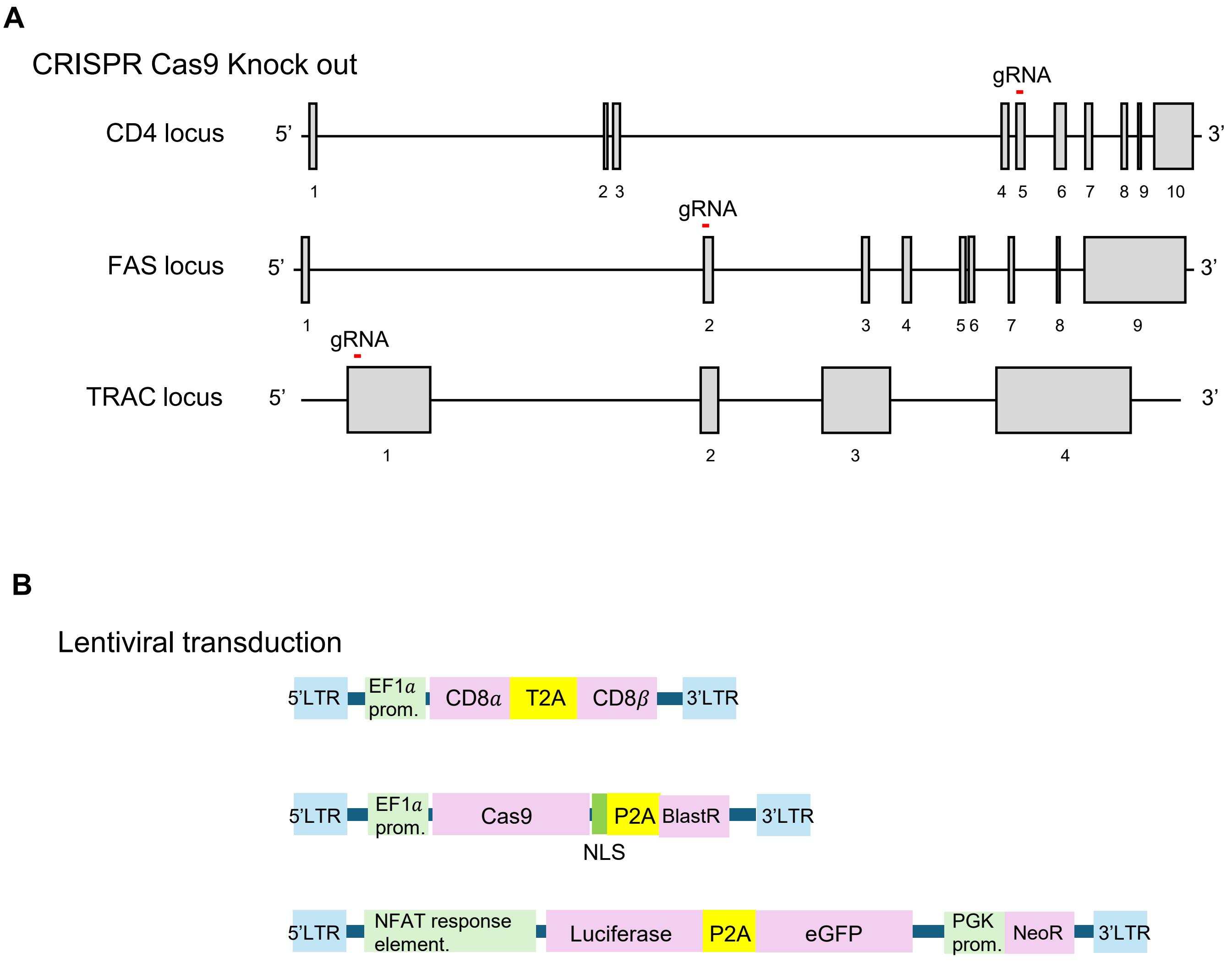

Figure S1. Gene editing targets and lentiviral constructs for engineering reporter T cells.
(A) Genomic loci of CD4, FAS, and TRAC in Jurkat cells, with the positions of gRNA target sites indicated.
(B) Schematic representation of lentiviral constructs encoding CD8α, CD8β, Cas9, and the NFAT–Luc–P2A–eGFP reporter cassette.

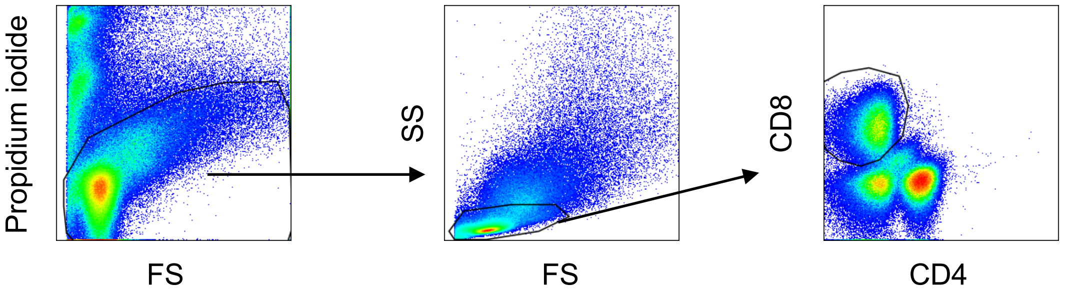

Figure S2. Gating strategy for CD8$^+$ tumor infiltrating T cell sorting from LK117 tumor
Single-cell suspensions were gated for live (propidium iodide$^-$) and lymphocytes (FSC/SSC). CD4$^-$ CD8$^+$ cells were then sorted.

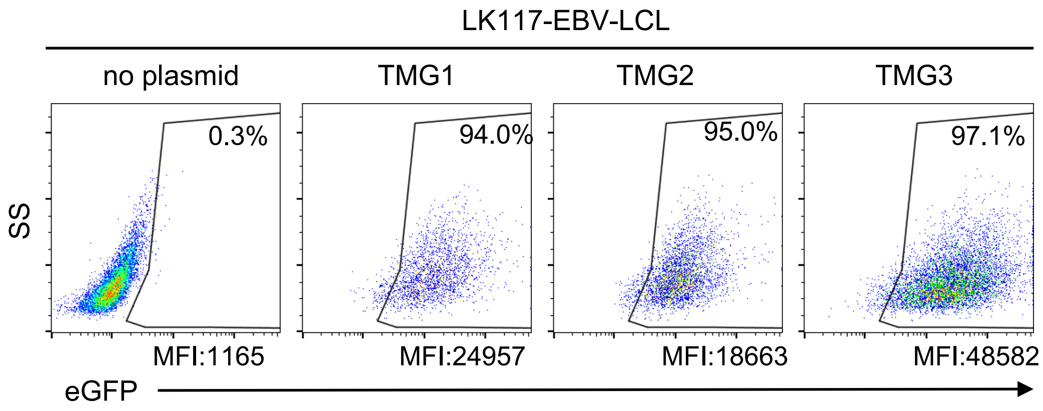

Figure S3 eGFP expression in TMG-transfected EBV-LCLs after sorting.
Autologous EBV-LCLs were electroporated with LK117 TMG constructs. Three days later, $eGFP^+$ cells were enriched using a cell sorter. eGFP was assessed prior to co-culture with reporter T cells. Dot plots show eGFP expression. Numbers indicate the frequencies of $eGFP^+$ cells and the mean fluorescence intensity (MFI).

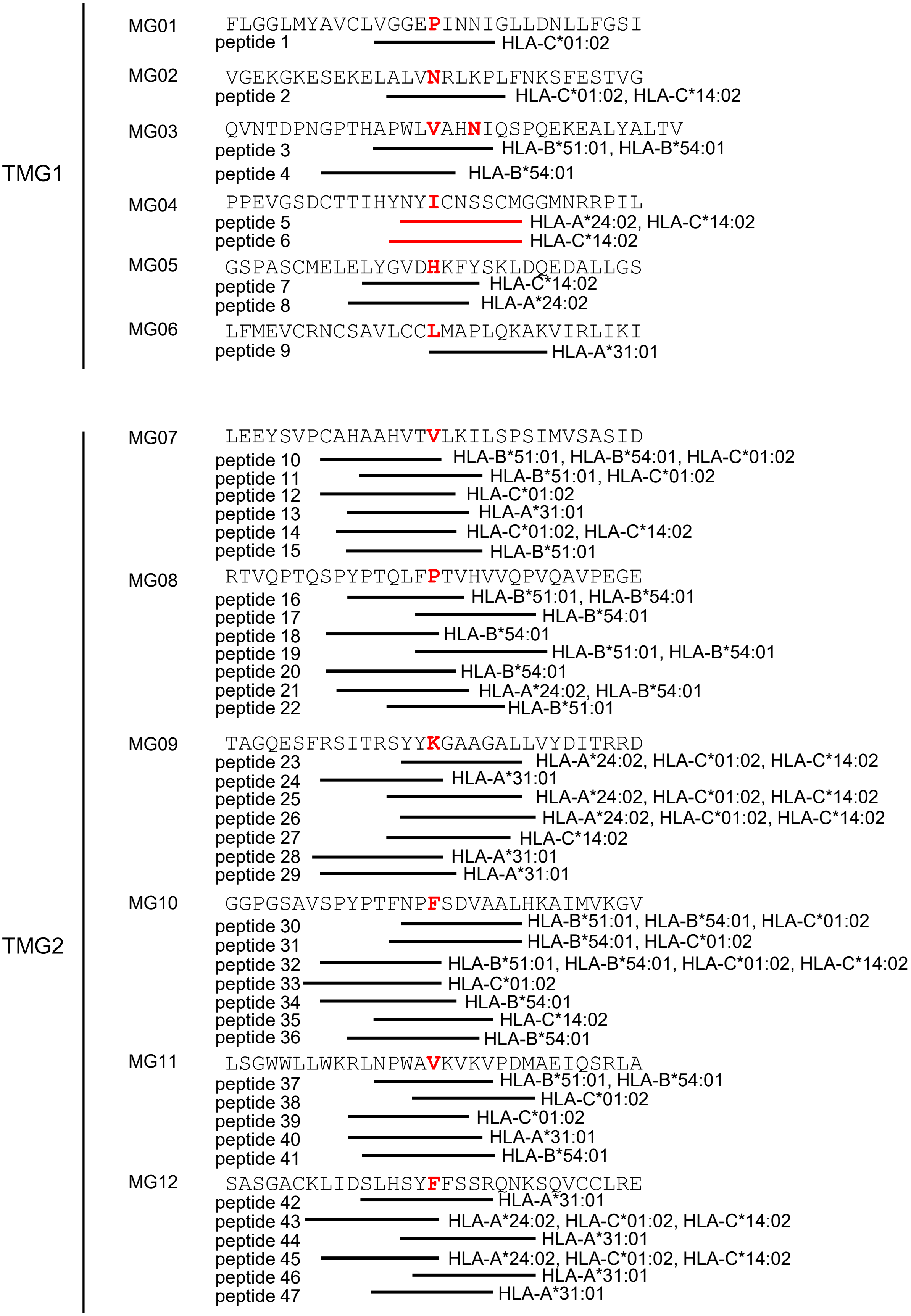
TMG1
MG01
FLGGLMYAVCLVGGEPINNIGLLDNLLFGSI
peptide 1
HLA-C*01:02
MG02
VGEKGKESEKELALVNRLKPLFNKSFESTVG
peptide 2
HLA-C*01:02, HLA-C*14:02
MG03
QVNTDPNGPTHAPWLVAHNIQSPQEKEALYALTV
peptide 3
HLA-B*51:01, HLA-B*54:01
peptide 4
HLA-B*54:01
MG04
PPEVGSDCTTIHYNYICNSSCMGGMNRRPIL
peptide 5
HLA-A*24:02, HLA-C*14:02
peptide 6
HLA-C*14:02
MG05
GSPASCMELELYGVDHKFYSKLDQEDALLGS
peptide 7
HLA-C*14:02
peptide 8
HLA-A*24:02
MG06
LFMEVCRNCSAVLCCLMAPLQKAKVIRLIKI
peptide 9
HLA-A*31:01
TMG2
MG07
LEEYSVPCAHAAHVTVLKILSPSIMVSASID
peptide 10
HLA-B*51:01, HLA-B*54:01, HLA-C*01:02
peptide 11
HLA-B*51:01, HLA-C*01:02
peptide 12
HLA-C*01:02
peptide 13
HLA-A*31:01
peptide 14
HLA-C*01:02, HLA-C*14:02
peptide 15
HLA-B*51:01
MG08
RTVQPTQSPYPTQLFPTVHVVQPVQAVPEGE
peptide 16
HLA-B*51:01, HLA-B*54:01
peptide 17
HLA-B*54:01
peptide 18
HLA-B*54:01
peptide 19
HLA-B*51:01, HLA-B*54:01
peptide 20
HLA-B*54:01
peptide 21
HLA-A*24:02, HLA-B*54:01
peptide 22
HLA-B*51:01
MG09
TAGQESFRSITRSYYKGAAGALLVYDITRRD
peptide 23
HLA-A*24:02, HLA-C*01:02, HLA-C*14:02
peptide 24
HLA-A*31:01
peptide 25
HLA-A*24:02, HLA-C*01:02, HLA-C*14:02
peptide 26
HLA-A*24:02, HLA-C*01:02, HLA-C*14:02
peptide 27
HLA-C*14:02
peptide 28
HLA-A*31:01
peptide 29
HLA-A*31:01
MG10
GGPGSAVSPYPTFNPFSDVAALHKAIMVKGV
peptide 30
HLA-B*51:01, HLA-B*54:01, HLA-C*01:02
peptide 31
HLA-B*54:01, HLA-C*01:02
peptide 32
HLA-B*51:01, HLA-B*54:01, HLA-C*01:02, HLA-C*14:02
peptide 33
HLA-C*01:02
peptide 34
HLA-B*54:01
peptide 35
HLA-C*14:02
peptide 36
HLA-B*54:01
MG11
LSGWWLLWKRLNPWAVKVKVPDMAEIQSRLA
peptide 37
HLA-B*51:01, HLA-B*54:01
peptide 38
HLA-C*01:02
peptide 39
HLA-C*01:02
peptide 40
HLA-A*31:01
peptide 41
HLA-B*54:01
MG12
SASGACKLIDSLHSYFFSSRQNKSQVCCLRE
peptide 42
HLA-A*31:01
peptide 43
HLA-A*24:02, HLA-C*01:02, HLA-C*14:02
peptide 44
HLA-A*31:01
peptide 45
HLA-A*24:02, HLA-C*01:02, HLA-C*14:02
peptide 46
HLA-A*31:01
peptide 47
HLA-A*31:01

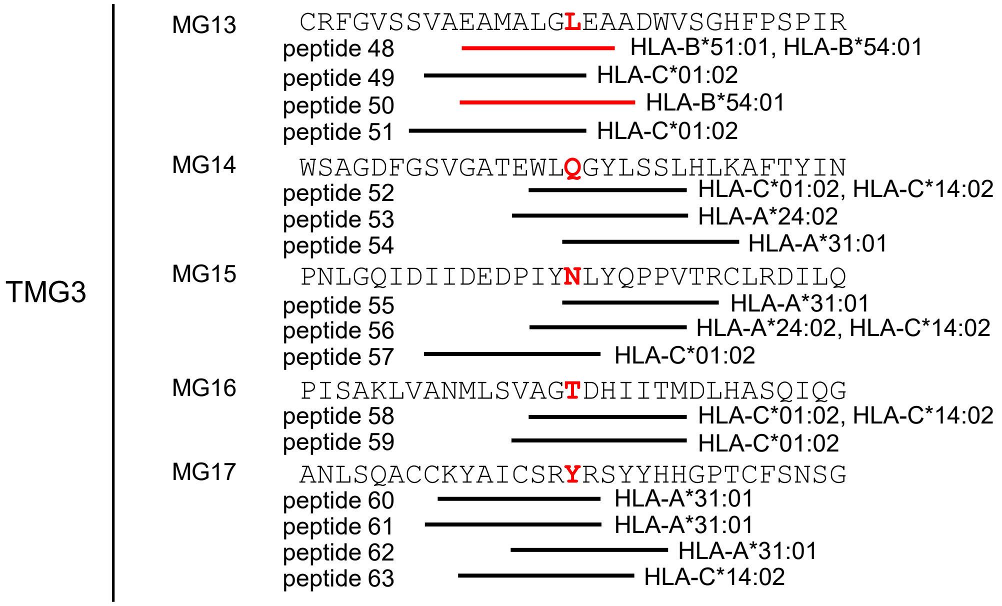

Figure S4. Tandem minigene (TMG) design and coverage of candidate HLA class I-binding peptides

This figure illustrates that each minigene (MG) encodes a sequence window that covers multiple candidate peptides spanning the somatic mutation. Shown are amino-acid sequences for TMG1 (MG01- MG06), TMG2 (MG07- MG12), and TMG3 (MG13- MG17). The red bold residue marks the mutated position. Black horizontal bars denote the 9- or 10-mer peptides predicted as candidates based on in-silico HLA class I binding prediction (Predicted HLA alleles are shown to the right of each bar.). Peptide IDs in the figure correspond to those used in the screening workflow; the peptide sequences, lengths, and source gene/mutation are provided in Table S8.

Red bars indicate neoantigen peptides identified as functional hits by NeoPAIR-T.

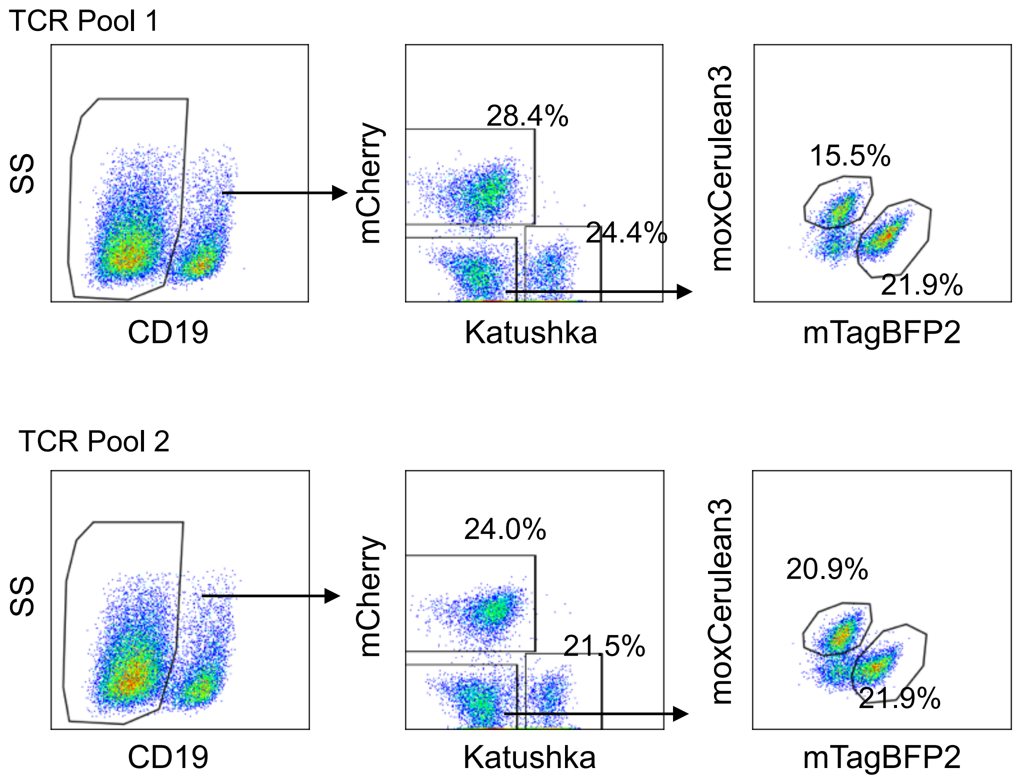

Figure S5. Frequencies of individual reporter T cell populations in pooled cells
Reporter T cells were prepared as described in the legend for Figure 7. Expression of mCherry, Katushka, moxCerulean3, and mTagBFP2 was analyzed by flow cytometry within the CD19- population for both T cell receptor (TCR) Pool 1 and Pool 2. Numbers indicate the percentage of each fluorescent subset among CD19^{-} cells.
